# Analysis of weighted gene co-expression network of triterpenoid-related transcriptome characteristics from different strains of *Wolfiporia cocos*

**DOI:** 10.1038/s41598-021-97616-6

**Published:** 2021-09-14

**Authors:** Guiping Zeng, Zhong Li, Zhi Zhao

**Affiliations:** 1 Guizhou University, Guiyang, 550025 China; 2 Guizhou Key Laboratory of Propagation and Cultivation on Medicinal Plants, Guizhou University, Guiyang, 550025 China

**Keywords:** Genetics, Molecular biology, Physiology

## Abstract

The fungus *Wolfiporia cocos* has wide-ranging and important medicinal value, and its dried sclerotia are used as a traditional Chinese medicine. Modern studies have shown that triterpenoid, the active ingredient of *W. cocos*, have a variety of pharmacological effects. The aim of our research was to determine the key genes related to triterpenoid biosynthesis, which may be useful for the genetic modification of cell-engineered bacteria for triterpenoid biosynthesis. In this study, two monospore strains, DZAC-WP-H-29 (high-yielding) and DZAC-WP-L-123 (low-yielding), were selected from the sexually propagated offspring of strain 5.78 of *W. cocos*, and the mycelia were cultured for 17, 34, and 51 days, respectively. Weighted gene co-expression network analysis (WGCNA) method was used to analyze transcriptional expressions. The results show that eight core genes (*ACAT1-b*, *hgsA*, *mvd1*, *SQLE*, *erg6*, *TAT*, *erg26*, and *erg11*) are associated with the triterpenoid synthesis pathway, and *Pm20d2* and *norA* outside the pathway may be important genes that influence the biosynthesis and accumulation of *W. cocos* triterpenoid. The biosynthesis of *W. cocos* triterpenoid is closely related to the expression of sterol metabolic pathway genes. The role of these genes in triterpenoid synthesis complements our knowledge on the biosynthesis and accumulation of *W. cocos* triterpenoid, and also provides a reference for the target gene modification of engineered bacteria for the fermentation production of triterpenoid.

## Introduction

The dried sclerotia of *Wolfiporia cocos* (Schwein.) Ryvarden & Gilb are used as a traditional Chinese medicine. *W. cocos* is mild in nature, sweet, and light in function. *W. cocos* can be used in the treatment of disease caused by the meridian of the heart, lungs, spleen, and kidneys. It has the effects of diuresis, invigorating and tonifying the spleen, tranquilizing the heart, and soothing the spirit. It is used for urinary problems, phlegm, dizziness and palpitations, spleen deficiency, loose stools, restlessness, and insomnia ^1^. *W. cocos* is frequently used as a medicine and as a food in Chinese medicine; there is a saying, "nine out of ten prescriptions require *W. cocos*". Therefore, *W. cocos* has a wide-ranging and important role in medical practice.

The main active components of *W. cocos* are polysaccharides and triterpenoids ^2^. Triterpenoid saponins are composed of hydrophobic triterpenoid glycosides and one or more hydrophilic glycosides ^3^. Triterpene saponins are secondary metabolites of plants and participate in the regulation of plant communication, defense, and sensory functions^4^. Modern studies have shown that *W. cocos* triterpenoids have immunoregulatory ^5,6^, antitumor ^7,8^, anti-inflammatory ^9,10^, diuretic ^11,12^, antioxidant ^13,14^, hepatoprotective ^15^, and anticonvulsant effects ^16,17^, among others. They are used as herbicides and insecticides in agriculture ^18,19^. Triterpenoid saponins are amphiphilic compounds that can form stable soap-like foams in aqueous solutions and are used in the detergent and cosmetics industries ^20^. Therefore, triterpenoid saponins play important roles in medicine, agriculture and the chemical industry.

Triterpenoid saponins are mainly extracted from plants, which generally take a long time to cultivate and produce a low yield. Compared with plants, large-scale microorganism fermentation has the advantages of fast growth, land saving, and high cost-effectiveness. Microbial production of triterpenoid saponins is considered as a promising alternative to traditional supply methods. Although triterpenoid saponins have been synthesized successfully in microbial hosts^21,22^, there are still many problems in increasing yields. The biosynthesis pathway of most triterpenoid saponins is not clear. Some key enzymes in plants are difficult to express in microbial hosts. Metabolic flux through the heterogeneous pathway is generally low. Some triterpene saponins are toxic to microbial cells ^23^. *W. cocos* is a fungus that synthesizes triterpenoid by itself. The content of triterpenoid in its hyphae is much higher than that in its sclerotia. *W. cocos* is a natural cell factory to produce triterpenoid saponins with natural resistance to the toxicity of triterpenoid on cells.

Weighted gene co-expression network analysis (WGCNA) is a method that analyzes the expression patterns of multiple sample genes, clusters the expression patterns of similar genes, and analyzes the correlation between a module and a specific trait or phenotype. Therefore, WGCNA is widely used in the study of diseases and other traits for genetic correlation analysis. The WGCNA algorithm ^24^ first assumes that the gene network obeys a scale-free distribution, defines the correlation matrix of gene expression and the adjacency function of gene network formation, calculates the otherness coefficient of different nodes, and then constructs a hierarchical clustering tree on the basis of the calculation results. Different branches of the eigengene dendrogram represent different gene modules; the degree of gene co-expression within the same module is high, but the degree of gene co-expression between different modules is low. Finally, the correlation between each module and a specific phenotype or disease is explored to identify the target genes for disease treatment and gene networks.

Both WGCNA and Short Time-Series Expression Miner (STEM) ^25^ are gene co-expression analysis methods. Compared with STEM analysis, WGCNA has the following advantages. (1) In terms of clustering method, it uses a weighted gene co-expression strategy (no scale distribution), which is more consistent with biological phenomena. (2) The interaction relationship between genes can be presented, and the hub genes at the center of the co-expression network can be found. (3) It is suitable for large sample sizes, and the more samples the better. By contrast, if a STEM analysis is carried out for more than 5 points, the results will be very complicated and the accuracy will be reduced. STEM analysis can only be carried out for 8 points at most. (4) Correlation with phenotype is possible; correlation analysis between module characteristic values, hub genes, and specific traits and phenotypes can be carried out to analyze biological problems more accurately.

In order to reveal the key genes and regulatory factors related to triterpenoid biosynthesis in *W. cocos* mycelia, this study selected two strains with significantly different triterpenoid contents as materials and performed hypha transcriptome analysis at three different culture times. WGCNA was used for comprehensive analysis, thereby laying a theoretical foundation for improving the triterpenoid biosynthesis yield of *W. cocos*.

## Materials and methods

### Biomaterials and culture methods

Both the high-yielding (DZAC-Wp-H-29) and low-yielding (DZAC-Wp-L-123) triterpenoid strains were derived from the sexually reproduced progeny strain 5.78 of *W. cocos* (purchased from the Institute of Microbiology, Chinese Academy of Sciences, Beijing, China, and stored in a refrigerator at − 80 °C at the Institute of Fungal Resources, Guizhou University). For the *W. cocos* potato dextrose agar (PDA) medium (no. 17 medium, Institute of Microbiology, Chinese Academy of Sciences), potatoes were washed, peeled, and cut into pieces, and 200 g of potatoes was put into 1000 mL of water, boiled for 30 min, then filtered by gauze. The filtrate was mixed with 1000 mL distilled water with 20 g glucose, 1 g KH_2_PO_4_, 0.5 g MgSO_4_·7H_2_O, 10 mg VB_1_, and 18 g agar at natural pH. Mycelia were cultured for 17, 34, and 51 d at 25 °C in the dark, quickly frozen in liquid nitrogen, then, stored in a refrigerator at − 80°C^26^.

### Colorimetry measurement of total triterpenoid

Colorimetric determination of total triterpenoid of *W. cocos* was modified with reference to Liu et al. ^27^. First, 0.05 g of dry mycelium powder (60 mesh) was placed in a 2 mL centrifuge tube, and 1.5 mL anhydrous ethanol was added. After ultrasonic extraction for 15 min, followed by centrifugation at 10,000 r/min for 5 min, the supernatant was placed in a 5 mL volumetric flask. Then, 1.5 mL anhydrous ethanol was again added to the centrifuge tube. After ultrasonic treatment for 15 min and centrifugation at 10,000 r/min for 5 min, the supernatant was taken and merged into the 5 mL volumetric flask, and anhydrous ethanol volume was added to the flask. Then, 2 mL of extract was placed in a test tube, volatilized at 50 °C, and cooled. Then, 0.2 mL of 5% vanillin in glacial acetic acid and 1 mL of perchloric acid were added and mixed in. The mixture was bathed in 70 °C water for 20 min, then removed from the water bath and cooled to room temperature, and 5 mL of anhydrous ethanol was added and mixed in. Then, 200 μL of mixed liquor was taken for absorbance measurement at 560 nm for 10–25 min; the reference substance was oleanolic acid^26^.

### RNA extraction and quantification analysis

Because *W. cocos* hyphae are rich in polysaccharides, total RNA was extracted by total RNA extraction auxiliaries and RNAiso Plus (Takara, China Bao Biological Engineering (Dalian) Co., Ltd. Dalian, China.), DNA pollution was removed by adding RNase-free DNase I, and three biological repeats were carried out. Total RNA was detected on 1% agarose gel and examined by NanoDrop ND2000 spectrophotometer (NanoDrop Technologies, Wilmington, DE, USA). The RNA integrity number (RIN) values (> 8.0) of these samples were evaluated by Agilent 2100 Bioanalyzer (Santa Clara, CA, USA). The purity, concentration, and integrity of total RNA samples were qualified through testing and evaluation, then the samples were prepared for use^26^.

### Construction and sequencing of cDNA library

First, mRNA was isolated from total RNA with Oligo (dT) beads, then broken into short fragments with fragment buffer. Then, short fragments were reverse transcribed into the first-strand cDNA with a random primer, and the second-strand cDNA was synthesized with DNA polymerase I, RNase H, dNTP, and buffer solution. The cDNA fragments were purified with 1.8× Agencourt AMPure XP Beads and end-repaired, and poly (A) was added and ligated to Illumina sequence adapters. The ligation products were size-selected by agarose gel electrophoresis, PCR-amplified, and sequenced using Illumina HiSeqTM 4000 by Gene Denovo Biotechnology Co. (Guangzhou, China)^26^.

### Sequence assembly and functional annotations

Reads obtained from the sequencing machines included dirty reads containing adapters or low-quality bases, which would affect the assembly and analysis. Thus, read adapters, unknown nucleotides, and low-quality reads were removed to obtain clean, high-quality reads. For filter reads using one’s own scripts, the parameters of data-processing steps are as follows: (1) Remove reads containing adapters. (2) Remove reads with N (unknown base) with a ratio greater than 10%. (3) Remove low-quality reads (bases with mass value Q ≤ 20, here accounting for more than 40% of reads). (4) Obtain clean reads.

De novo transcriptome assembly was carried out with the Trinity short reads assembling program ^28^. The software parameters were as follows: kmer size = 31, min kmer cov = 12; all other nonimportant parameters were default values. Clean reads were aligned with reference sequences to obtain an alignment rate with Bowtie2 short reads alignment software ^29^. The software parameters were the default parameters.

Basic annotation of unigenes includes protein functional, pathway, Cluster of Orthologous Groups of proteins (COG/KOG) functional and GO (Gene Ontology) annotation. To annotate the unigenes, we used the BLASTx program (http://www.ncbi.nlm.nih.gov/BLAST/) with an E-value threshold of 1 × 10^−5^, giving priority to the National Center for Biotechnology Information (NCBI) non-redundant protein (Nr) database (http://www.ncbi.nlm.nih.gov), the Swiss-Prot protein database (http://www.expasy.ch/sprot), the KEGG (Kyoto Encyclopedia of Genes and Genomes) database^30^ (http://www.genome.jp/kegg), the COG/KOG database (http://www.ncbi.nlm.nih.gov/COG) and Plant Transcription Factor Database (http://plntfdb.bio.uni-potsdam.de/v3.0/). Protein functional annotations could be obtained according to the best alignment results. Finally, ESTScan software ^31^ was used to predict the coding region of unigenes that could not be compared with the above protein libraries, and the nucleic acid sequence (sequence direction 5′ → 3′) and amino acid sequence of the coding region were obtained.

GO annotation information of unigenes was analyzed by Blast2GO software according to the Nr annotation information ^32^, then functional classification of unigenes was performed by WEGO software ^33^.

### Unigene expression differential analysis

Unigene expression was calculated and normalized to RPKM ^34^. The formula is RPKM = (1,000,000 × C)/(N × L/1000)

$${\text{RPKM}} = \left( {{1},000,000 \, \times {\text{ C}}} \right)/\left( {{\text{N }} \times {\text{ L}}/{1}000} \right)$$

where RPKM is the expression of unigene A, C is the number of reads that are uniquely mapped to unigene A, N is the total number of reads that are uniquely mapped to all unigenes, and L is the length (base number) of unigene A. Concordant PE read alignments were used to normalize the calculation.

Difference analysis based on edgeR ^35^ was implemented by the R package. Normalization uses the calcNormFactors function embedded in edgeR. Gene dispersion uses the estimateTagwiseDisp function. Differentially expressed genes (DEGs) were those with false discovery rate (FDR) < 0.05 and |log_2_FC| ≥ 1. The calculation method of FDR ^36^ is based on the method of Benjamini and Hochberg. The formula is FDR = p × (m/k), where p is the p-value, m is the number of inspections, and k is the rank of the inspection p-values among all p-values (from small to large).

### RT-qPCR validation

RT-qPCR (real-time quantitative polymerase chain reaction) specific primers were designed with Beacon Designer 7.9 (Beijing Biological Technology Co., Ltd. Beijing, China) (Supplementary Table S1). The first strand of cDNA was obtained by reverse transcription with Aidlab’s reverse transcription kit (TUREscript 1st Strand cDNA Synthesis Kit, Aidlab Biotechnologies Co.,Ltd. Beijing, China). RT-qPCR was conducted by using the qTOWER 2.2 PCR System (Jena, Germany) and 2 × SYBR® Green PCR Master Mix (DBI). Each reaction was performed in a total reaction mixture volume of 10 μL containing 1 μL of first-strand cDNA as a template. The amplification program was as follows: 3 min at 95 °C; 40 cycles of 10 s at 95 °C, 30 s at 58 °C, and 45 s at 72 °C; and finally 10 min at 72 °C. All RT-qPCR experiments were repeated three times, with three technical repeats for each experiment. Expression levels of candidate genes were determined using the 2^−∆∆*C*t^ method^26^. Expression levels were normalized against the reference gene pab1 (unigene0013050).

### Weighted gene co-expression network analysis (WGCNA)

The R language package was used for analysis ^24^. Firstly, the low quality data were filtered, then the modules were divided. The Power value was 0.8, the similarity was 0.7, the minimum number of genes in a module was 50, and the rest were default parameters.

### Statistical analysis

SPSS Statistics software was used for basic calculations. A single factor ANOVA in comparative mean analysis was used for significance test. Standardized data are obtained through descriptions in descriptive statistical analysis. The principal component and correlation analyses were conducted with the R-language package (R 3.4.3 2017) (http://www.r-project.org/). Select the default parameters to run. Graph Pad Prism7.0 was used for histograms, heat maps, and correlation graph. Both the histogram and the heat map were obtained by setting up the grouped. The correlation graph was obtained by inputting the data into the XY table for correlation and linear regression analysis. Cytoscape3.7.1 was used to map the gene–gene co-expression network. First, input a file containing the weight value between gene and gene, and an attribute file containing symbol, type and the connectivity value of genes in the module. Adobe Illustrator CS6 was used for illustration.

## Results

### Analysis of total triterpenoid contents and genes in high-yielding and low-yielding strains

A colorimetric method ^27^ was used to determine the content of triterpenoid in *W. cocos.* There were very significant differences between the two strains (Supplementary Figure S1). The results indicated that differences in gene expression at different culture times may lead to differences in the synthesis and final accumulation of triterpenoid secondary metabolites ^26^.

Transcriptome sequencing results (Table 1) and quality evaluation (Supplementary Table S1) showed that the assembly quality of sequencing was good. Real-time quantitative polymerase chain reaction (RT-qPCR) was conducted on 12 randomly selected genes (Supplementary Table S2) with TUBB2 as the internal reference gene. In Supplementary Figure S2, each point represents a value of fold change of expression level at d34 or d51 comparing with that at d17 or d34. Fold-change values were log 10 transformed. The results showed that the gene expression trend was consistent in transcriptome sequencing and RT-qPCR experiments, and the data showed a good correlation (r = 0.530, *P* < 0.001, Supplementary Figure S2). For each gene, the expression results of RT-qPCR showed a similar trend to the expression data of transcriptome sequencing (Supplementary Figure S3). Furthermore, the transcriptome sequencing data in this study were shown to be reliable.

**Table 1:** Summary of splicing length distribution in RNA-seq.

Genes Num	GC content (%)	N50	Max length	Min length	Average length	Total assembled bases
16,879	54.817	2673	16,094	201	1602	27,053,706

**Figure 1:**
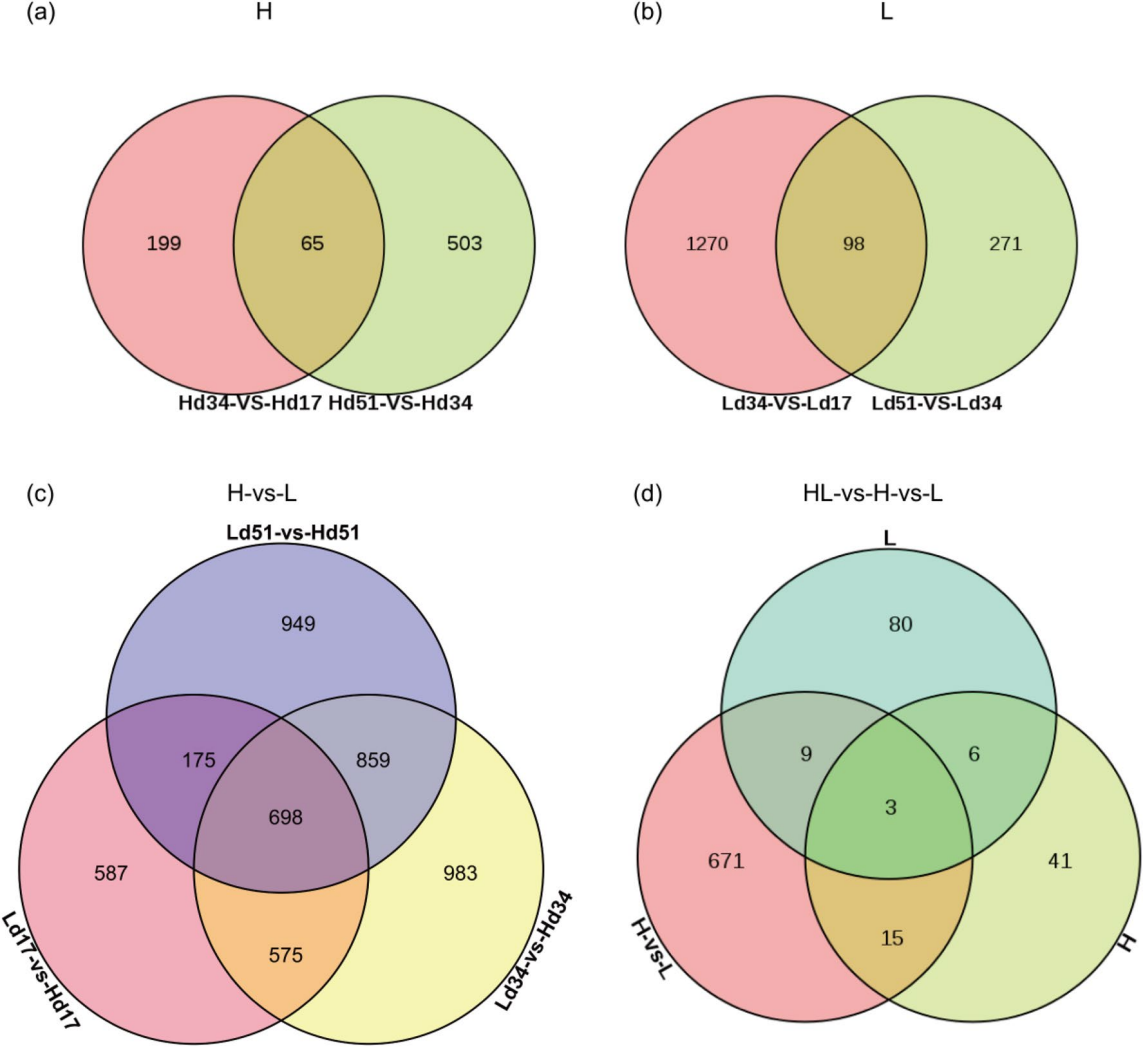
Venn diagrams of DEGs. (**a**) Venn diagram of DEGs during three cultivation periods of high-yielding strains; (**b**) Venn diagram of DEGs during three cultivation periods of the low-yielding strains; (**c**) Venn diagram of DEGs between high-yielding and low-yielding strains in three culture periods; (**d**) Venn diagram of DEGs in high-yielding strains, low-yielding strains, between high- and low-yielding strains.

Venn diagrams were created for the DEGs between high-yielding and low-yielding strains with three different culture times, respectively (Fig. 1). In the high-yielding (H) strain and low-yielding (L) strain, respectively, 65 and 98 overlapping DEGs were obtained (Fig. 1a,b), and 698 overlapping DEGs were obtained between H and L strains (Fig. 1c). 698 overlapping DEGs in three different culture times between H and L strains were significantly higher than those in the high-yielding and low-yielding strains, were 10.7 and 7.1 times, respectively. The DEGs between H and L strains cultured for 17 days, 34 days and 51 days were respectively 2035, 3115 and 2681, showing a trend of first increase and then decrease. The Venn diagram results of overlapping genes in the H strains, in the L strains, and between H and L strains showed that there was a large quantity of DEGs, while the number of overlapping genes was very few, at only 3 (Fig. 1d), and the number of overlapping DEGs between H and L strains was only 9. The Venn diagram results showed that the gene expression difference between the two strains was large, which was essentially different from the gene expression difference within strain due to different culture times.

Zeng et al. ^26^ used STEM to focus on genes whose expression trends were opposite in H and L strains with increasing culture time. The research results indicated that the accumulation of triterpenoid was affected by gene expression differences in high-yielding and low-yielding strains. However, according to the above Venn diagram analysis, the DEGs related to triterpenoid biosynthesis were different from those related to triterpenoid accumulation in the two strains that we tested. Therefore, the analysis of Zeng et al. ^26^ may have omitted the key genes affecting triterpenoid biosynthesis in the two strains.

### Modules related to triterpenoid biosynthesis revealed by WGCNA

**Figure 2:**
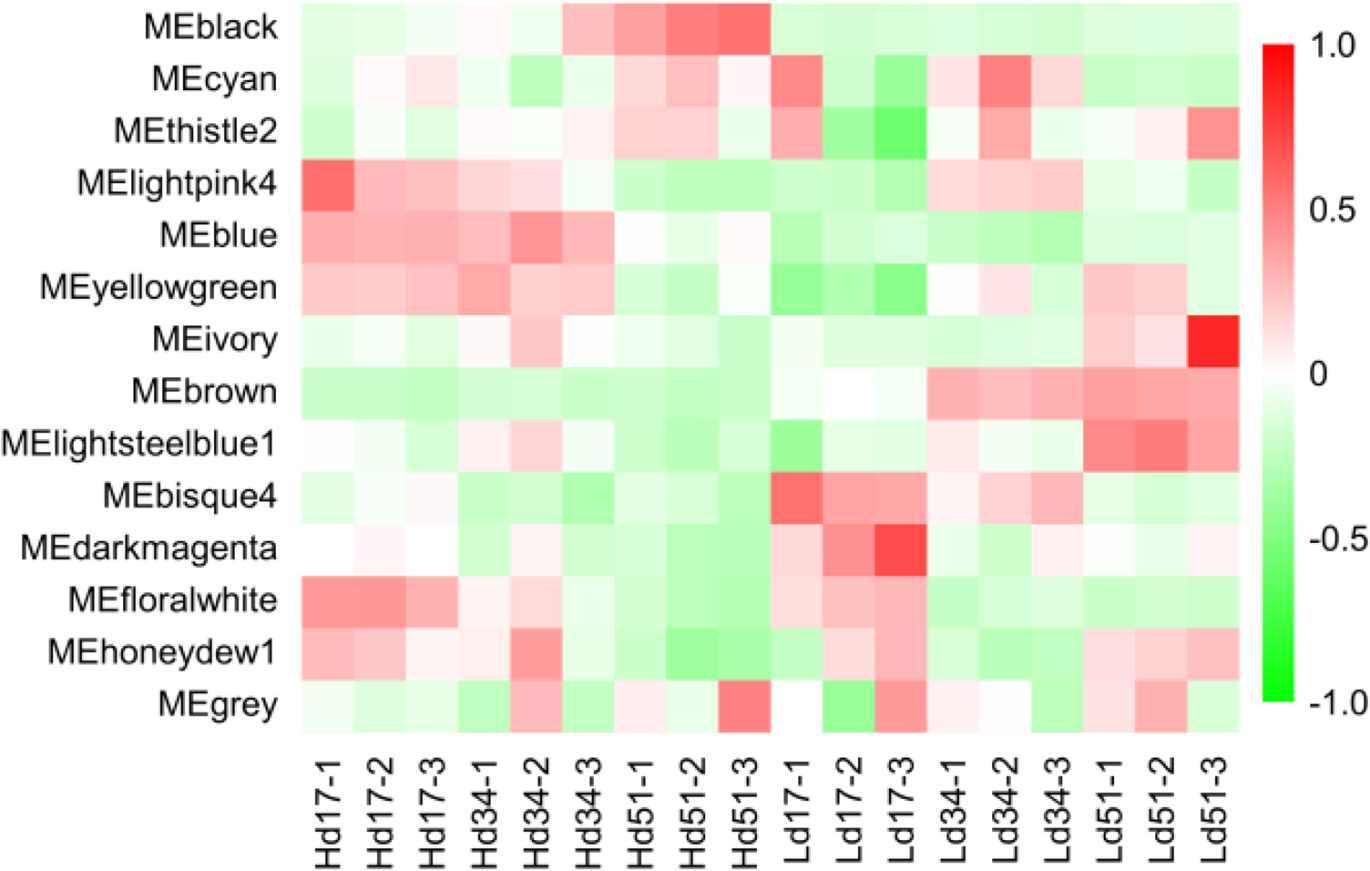
Expression pattern of module genes in each sample. The module characteristic value is the normalized value of the weighted composite value of all gene expressions in the module for each sample. Red represents high expression, and green represents low expression. (Graph Pad Prism7.0: https://www.graphpad.com/).

In order to identify the core genes of the regulatory network related to triterpenoid biosynthesis, we performed WGCNA on 18 samples’ transcriptome data. After data filtering, the Power value was selected as 8 to divide the modules, the similarity degree was selected as 0.7, the minimum number of genes in a module was 50, and 14 modules were finally obtained. The weighted composite value of all gene expression quantities in the module was used as the module characteristic value to draw the heat map of sample expression pattern (Fig. 2). It can be found that the gene expression quantities are significantly different between the high-yielding strain (H) and the low-yielding strain (L) in the three modules of blue, brown, and bisque4. The results of correlation analysis between two modules (Supplementary Figure S4) show that blue and brown, and blue and bisque4 are significantly negatively correlated, with correlation coefficients of − 0.7 and − 0.59, respectively. Brown and bisque4 are weakly correlated, with a correlation coefficient of 0.24.

### GO and KEGG enrichment analysis on blue, brown, and bisque4

**Figure 3:**
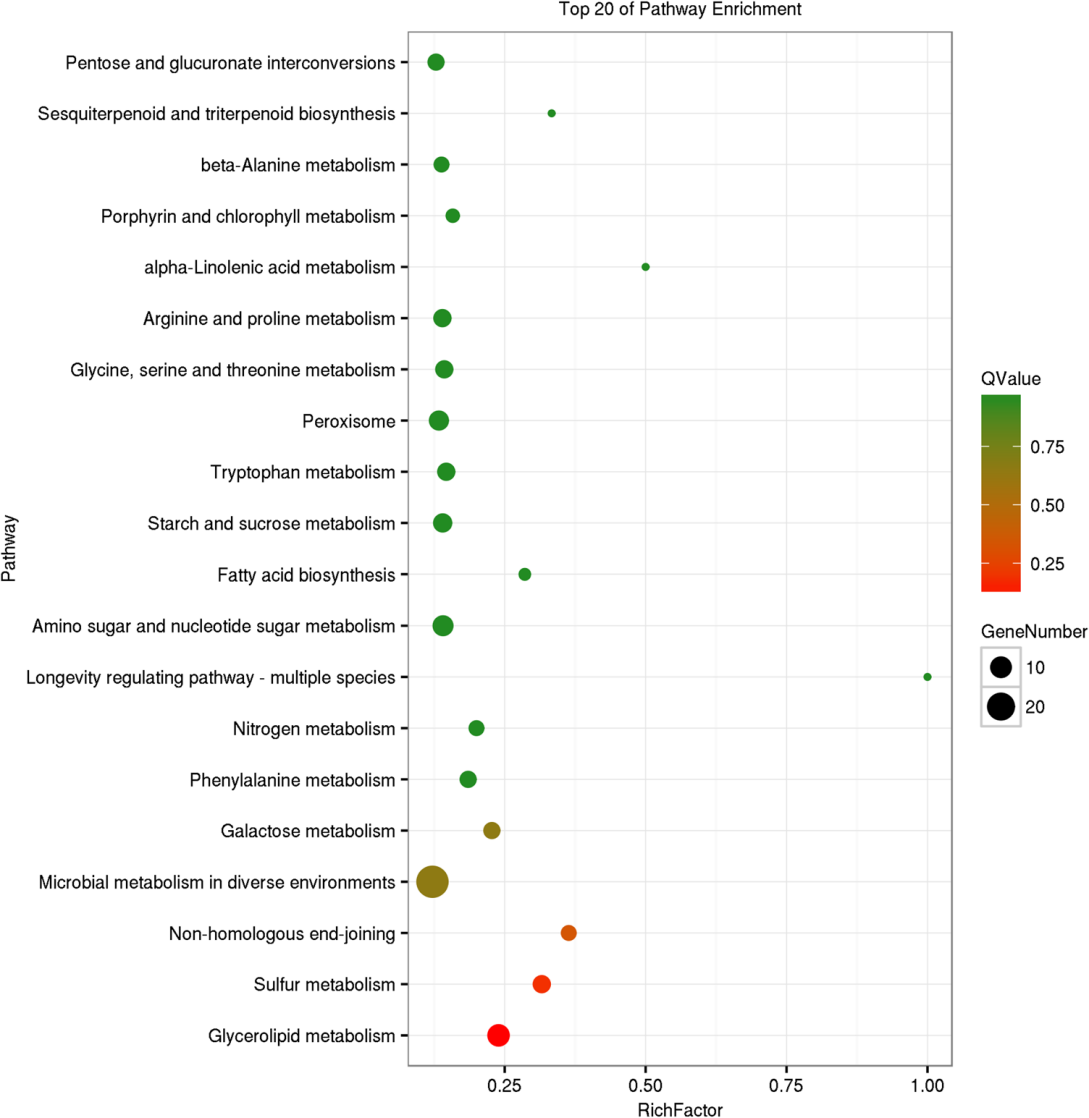
KEGG enrichment diagrams of gene of module brown. Vertical axis represents pathway entries, and Rich Factor on the horizontal axis refers to the ratio of the number of genes of DEGs in the pathway to the total number of genes of all genes in the pathway. The higher the Rich Factor value is, the higher enrichment degree is. Size of the circle corresponds to the number of enriched genes, and the larger the circle, the more genes there are. QValue is the *p*-value after multiple hypothesis test correction, which ranges from 0 to 1, corresponding to the gradual change of red to green. The closer it is to zero, the more red it is, and the more significant the enrichment is. This figure is plotted with the first 20 pathway of QValue of from smallest to largest.

**Figure 4:**
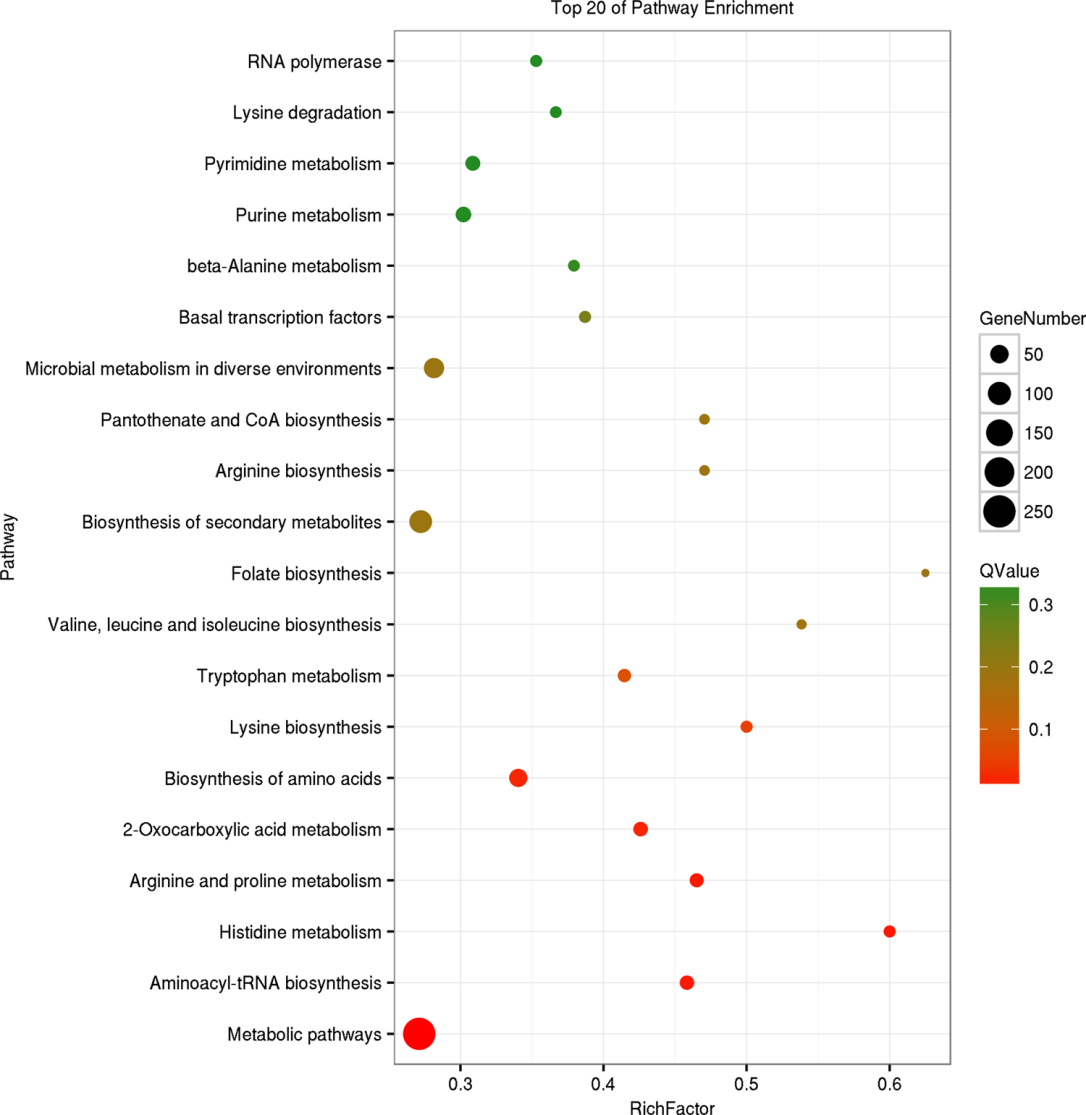
KEGG enrichment diagrams of gene of module blue. Vertical axis represents pathway entries, and Rich Factor on the horizontal axis refers to the ratio of the number of genes of DEGs in the pathway to the total number of genes of all genes in the pathway. The higher the Rich Factor value is, the higher enrichment degree is. Size of the circle corresponds to the number of enriched genes, and the larger the circle, the more genes there are. QValue is the *p*-value after multiple hypothesis test correction, which ranges from 0 to 1, corresponding to the gradual change of red to green. The closer it is to zero, the more red it is, and the more significant the enrichment is. This figure is plotted with the first 20 pathway of QValue of from smallest to largest.

**Figure 5:**
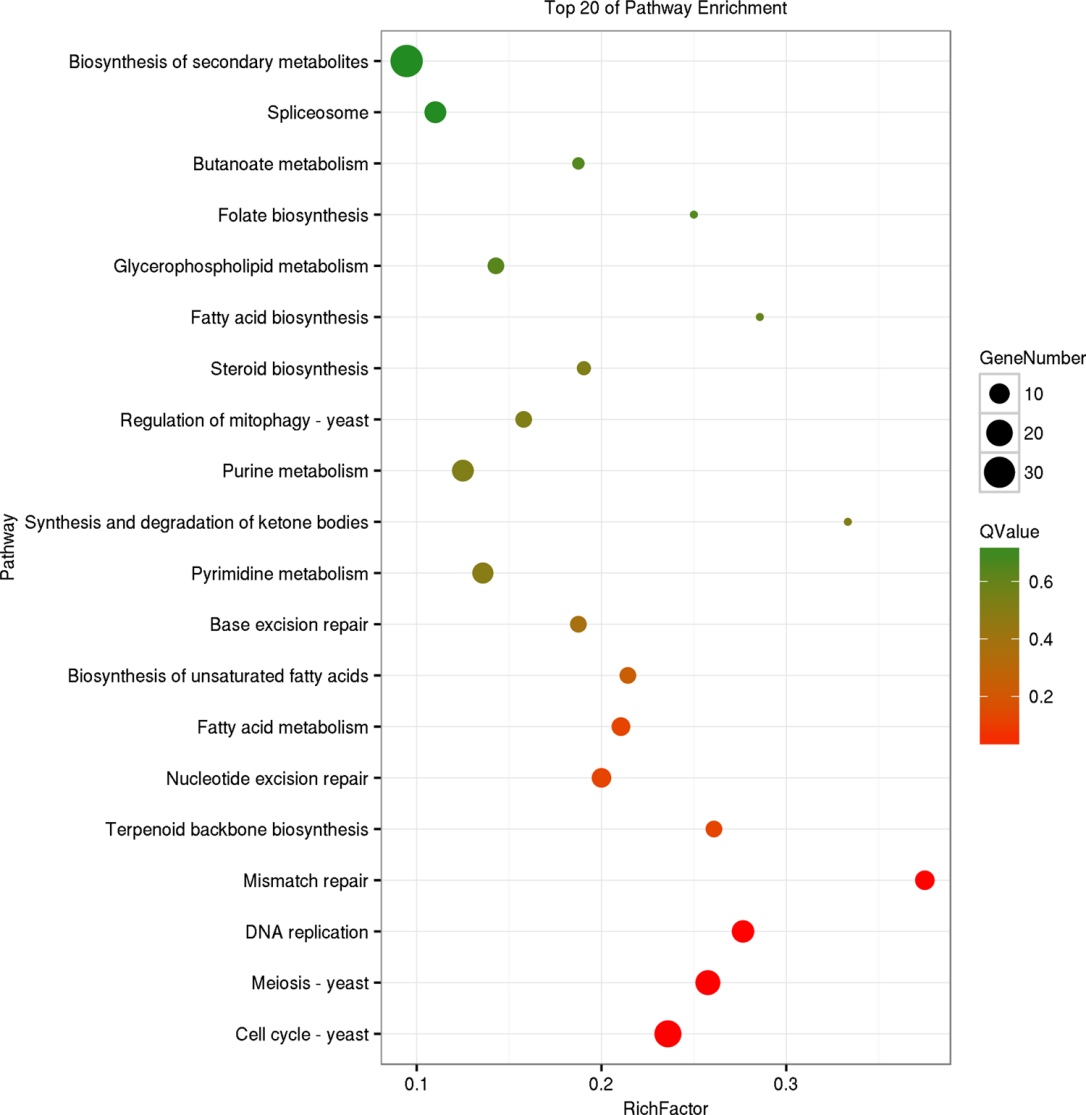
KEGG enrichment diagrams of gene of module bisque4. Vertical axis represents pathway entries, and Rich Factor on the horizontal axis refers to the ratio of the number of genes of DEGs in the pathway to the total number of genes of all genes in the pathway. The higher the Rich Factor value is, the higher enrichment degree is. Size of the circle corresponds to the number of enriched genes, and the larger the circle, the more genes there are. QValue is the *p*-value after multiple hypothesis test correction, which ranges from 0 to 1, corresponding to the gradual change of red to green. The closer it is to zero, the more red it is, and the more significant the enrichment is. This figure is plotted with the first 20 pathway of QValue of from smallest to largest.

GO enrichment analysis was carried out on genes in the three modules of blue, brown, and bisque4, respectively (Supplementary Figure S5). The results showed that genes in these three modules were mainly enriched in catalytic activity and binding in the molecular functions; metabolic processes, cellular processes, and single-organism processes in the biological processes; and cell and cell parts in the cellular component. The three modules had the same GO enrichment results, only the number of genes was different. Furthermore, KEGG enrichment results (Supplementary Table S3) for the three modules were not the same. The brown module (*P* < 0.05) was mainly enriched in the metabolic pathways of glyceride, sulfur, and galactose; non-homologous end joining; and microbial metabolism in diverse environments (Fig. 3). The blue module (*P* < 0.05) was mainly enriched in the metabolism and biosynthesis of various amino acids; metabolism of oxycarboxylic acid, and folate; biosynthesis of secondary metabolites, aminoacyl-tRNA, pantothenate, and CoA; microbial metabolism in diverse environments; basal transcription factors, etc. (Fig. 4). The bisque4 module (*P* < 0.05) was mainly enriched in the cell cycle; meiosis; DNA repair; mismatch repair; nucleotide excision repair; base excision repair; biosynthesis of terpenoid backbones, and unsaturated fatty acids; and fatty acid metabolism (Fig. 5). The KEGG enrichment results of the three modules were significantly different, which was consistent with the results of the module correlation analysis.

The genes related to triterpenoid anabolism in each module were selected according to KEGG annotation results of genes, and those genes with the above gene's expression correlation weight value among in the module were top 10 were selected (Supplementary Table S4). Those genes were selected for GO and KEGG enrichment. GO enrichment (Supplementary Figure S6) showed that these selected genes were mainly concentrated in catalytic activity and binding in the molecular functions; metabolic processes, cellular processes, and single-organism processes in the biological processes; and cell and cell parts in the cellular component. The enrichment of these three modules’ genes was still basically the same. Detailed GO information of these three modules’ genes is displayed in Supplementary Tables S5–7. KEGG enrichment results of genes related to triterpenoid biosynthesis in each module (Supplementary Table S8) showed that the brown module was only enriched in metabolism of amino sugars and nucleotide sugars. The blue module was mainly enriched in the metabolism and biosynthesis of various amino acids; biosynthesis of secondary metabolites; oxocarboxylic acid metabolism; microbial metabolism in diverse environments; and basal transcription factors. The bisque4 module was mainly enriched in biosynthesis of triterpenoid backbones, and unsaturated fatty acids; fatty acid metabolism; the cell cycle; and meiosis. Combined with the results of STEM analysis by Zeng et al. ^26^, a stable membrane structure may be necessary to maintain a high accumulation of triterpenoid in *W. cocos*, and the high accumulation capacity of triterpenoid in *W. cocos* may be related to the synthesis capacity of sterols. Only bisque4 of the three modules was significantly enriched in the biosynthesis of triterpenoid backbones and unsaturated fatty acids.

### Gene–gene correlation analysis for triterpenoid related genes of the three modules

Cytoscape was used to map the relationships according to the values of connectivity for the three modules’ genes related to triterpenoid biosynthesis. There are two core genes of sterol-4alpha-carboxylate 3-dehydrogenase (*erg26*) (unigene0006213) and lanosterol 14-alpha-demethylase (*erg11*) (unigene0015621) in the brown module (Supplementary Figure S7), which are both genes in the steroid biosynthetic pathway (KEGG annotation) and regulatory factors (PlnTFDB annotation). *Erg26* and *erg11* are regulated by multiple genes, respectively. *Erg26* is regulated by both the regulator *GIP* (Copia protein) (unigene0004283) and *OPT5* (Oligopeptide Transporter 5) (unigene0000595). *Erg11* is regulated by *Matk* (kinase-like protein) (unigene0006800) and *betA* (oxygen-dependent choline dehydrogenase) (unigene0011761). *ERG9* (farnesyl-diphosphate farnesyltransferase) (unigene0013210) has a weak correlation with *erg26*. *FDPS* (farnesyl-diphosphate synthase) (unigene0002741) is indirectly related to *erg26* and *erg11*. In addition, the three genes of *FACE1* (STE24 endopeptidase) (unigene0000435), *PST2* (unigene0001237), and *Fntb* (unigene0014799) are indirectly related in the module.

Except for *TAT* (tyrosine aminotransferase) (unigene0003146) with moderate connectivity, several other genes related to triterpenoid biosynthesis in the blue module (Supplementary Figure S8) have generally low connectivity. *TAT* has a direct or indirect relationship with *erg11* (unigene0015620), *ERG2* (C-8 sterol isomerase) (unigene0004578), *COQ2* (4-hydroxybenzoate polyprenyltransferase) (unigene0001642), *erg26* (unigene0007103), *FTA* (protein farnesyltransferase subunit beta) (unigene0010654), *ACAT* (sterol O-acyltransferase) (unigene0015643), *CAO2* (carotenoid oxygenase) (unigene0011352), and *COQ2* (unigene0001914), respectively. *Erg6* (sterol 24-C-methyltransferase) (unigene0004059) and *erg11* (unigene0012490) with low connectivity are associated with several different genes, respectively. *TAT* is regulated by four regulatory factors and multiple genes. The two regulatory factors *norA* (aryl-alcohol dehydrogenase) (unigene0005043) and *Pm20d2* (peptidase M20 domain-containing protein 2) (unigene0004261) in the module have high connectivity and are indirectly related to *TAT*.

In the bisque4 module (Supplementary Figure S9), except for *TAT* (unigene0012065), which has very low connectivity, the other 9 genes related to triterpenoid biosynthesis are correlated with each other and interlaced into a complex regulatory network. In particular, *erg6* genes (unigene0014738, unigene0014749), *SQLE* (squalene monooxygenase) (unigene0009035), *mvd1* (diphosphomevalonate decarboxylase) (unigene0001911), *ACAT1-b* (acetyl-CoA C-acetyltransferase) (unigene0014534), and *hgsA* (hydroxymethylglutaryl-CoA synthase) (unigene0000449) are five genes that have high connectivity and strong interactions, which are simultaneously regulated by regulators and multiple genes. The *erg6* (unigene0014738) gene is particularly important and interacts directly and indirectly with the four core genes *SQLE*, *mvd1*, *ACAT1-b*, and *hgsA*. Through the above correlation analysis of genes related to triterpenoid biosynthesis and metabolism, eight core genes (*ACAT1-b*, *hgsA*, *mvd1*, *SQLE*, *TAT*, *erg11*, *erg26*, and *erg6* genes) of the regulatory network were screened out from the three modules that may be related to triterpene anabolism.

### Screening of key genes in biosynthesis of triterpenoid in *W. cocos*

**Figure 6:**
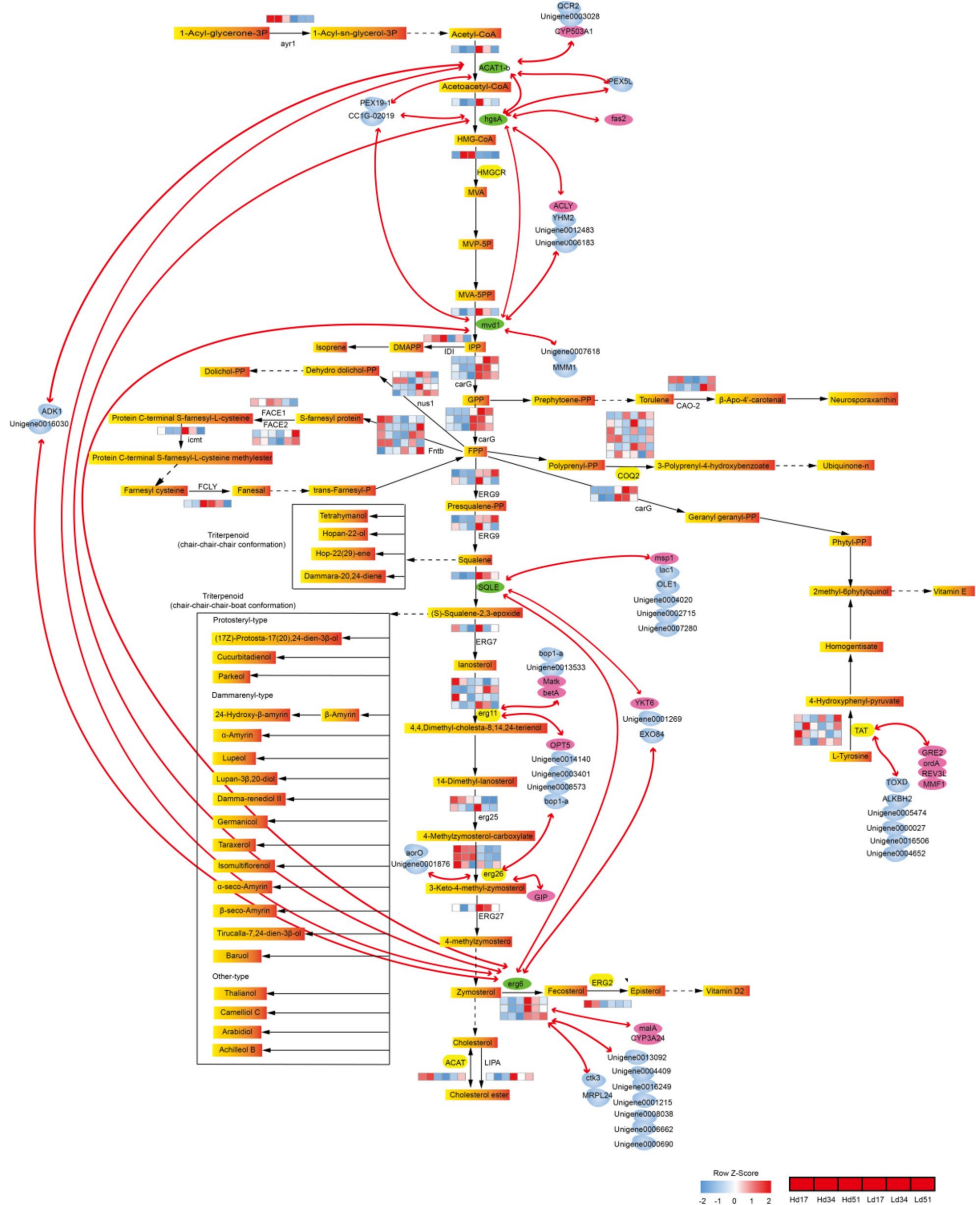
Illustration of the gene expression differences and relationships for triterpenoid-related genes in the two strains. Gradual orange rectangles represent the metabolite, enzyme abbreviation, and enzyme expression level for the three time points of the two strains marked on the bottom, left, or right of the arrow connecting two metabolites. Solid black arrows represent direct catalytic reactions, and dotted black arrows represent indirect catalytic reactions. Metabolites in solid black boxes are triterpene products of the same type catalyzed by different enzymes with the same precursor. Blue molecule shapes represent protease genes; purple elliptic traits represent regulators, marked with the gene abbreviation or ID. Red lines represent connecting correlations. The heat map of each gene’s reads per kilobase of transcript per million mapped reads (RPKM) value shows the standardized z-score. (Adobe Illustrator CS6: https://www.adobe.com/cn/products/illustrator.html).

KEGG was used for mapping the triterpenoid metabolic pathway. According to the co-expression relationship between genes in the above three modules, genes in the pathway are mapped to the metabolic pathway, while other genes are arranged outside the pathway (Fig. 6). Supplementary Figure S10 is standardized heat map of genes in Fig. 6. The eight core genes are located in the upstream and downstream pathways of triterpenoid biosynthesis. With the exception of *TAT*, the other seven core genes directly or indirectly interact with each other and are simultaneously affected by regulators or multiple protease genes.

In the bisque4 module, in the upstream of the biosynthesis of triterpenoid, three genes (*ACAT1-b*, *hgsA*, and *mvd1*) interact with each other and are closely related, and are also affected by the protease genes *PEX19-1* and *CC1G-02019*. *ACAT1-b* and *hgsA* are also affected by the protease gene *PEX5L*. Both *hgsA* and *mvd1* are influenced by the regulator *ACLY* and the protease gene *YHM2*. All three enzymes interact directly with *erg6*. *ACAT1-b*, the upstream core gene of the pathway, and *erg6*, the last downstream core gene of the pathway, are also co-acted upon by *ADK1* and unknown protein unigene0016030. *SQLE*, the core gene of the biosynthesis of triterpenoid, interacts directly with *erg6* and simultaneously interacts indirectly with *erg6* by the regulators *YKT6* (snare-like protein), *EXO84*, and unigene0001269. *SQLE* is also regulated by the regulator mitochondrial protein (*msp1*) and multiple protease genes. Network relationships show that the expression of *SQLE* is affected by many factors, especially the relationship with *erg6*. Two of the three *erg6* sequences are in the center of the network in the bisque4 module, affecting the expression of each core gene across the pathway. *Erg6* was co-expressed with several genes, including three regulators *YKT6*, *malA* (NADP-dependent malic enzyme), and cytochrome P450 (*CYP3A24*)) and 13 protein genes (Fig. 6, Supplementary Figure S9). The complexity of the network relationships indicates the complexity of core gene expression regulation. The core genes in the pathway regulate each other to affect their expressions and are also affected by many factors outside the pathway.

In the brown module, in the downstream of the biosynthesis of triterpenoid, *erg11* and *erg26* are jointly affected by the regulatory factor *OPT5* and multiple protease genes. *Erg11* is also affected by the regulatory factors *Matk* and *betA*, Ribosome biogenesis protein (*bop1-a*), and unknown protein unigene0013533. *Erg26* is affected by the regulator *GIP*, the protease gene *aorO*, and unigene0001876.

In the blue module, on the branches related to the biosynthesis of triterpenoid, the moderate connectivity of *TAT* is simultaneously affected by multiple regulators and protease genes, which have high connectivity. The network diagram shows that the regulatory pattern of *TAT* is very complex and many factors affect its expression. It is worth noting that *Pm20d2* and *norA* in the blue module have very high connectivity and are directly or indirectly related to multiple triterpenoid-related genes. They were also screened in the Short Time-series Expression Miner (STEM) analysis results of Zeng et al. ^26^ and were positively correlated with *erg26*, *ERG2*, and *TAT*; *Pm20d2* was negatively correlated with *erg11*.

## Discussion

In this study, the high-yielding DZAC-Wp-H-29 (H) and low-yielding DZAC-Wp-L-123 (L) strains of *W. cocos* with different total triterpenoid contents were screened from the sexual progeny of the same strain. The selection of materials and culture times avoided any background interference caused by different genetic bases or developmental stages of materials, making the research results more accurate and reliable. The weighted gene co-expression network analysis (WGCNA) method was used for analysis. Among the fourteen gene modules with similar expression patterns, three modules (bisque4, blue, and brown) were selected for further analysis according to the phenotypic differences in the triterpenoid contents of the two strains. The top 10 genes with the highest connectivity values in relation to triterpenoid-related genes in each module were selected, and a network diagram was built according to the gene connectivity relationships in each module. Five core genes (*ACAT1-b*, *hgsA*, *mvd1*, *SQLE*, and two *erg6* genes) in the bisque4 module constituted a complex network of direct and indirect effects, with *erg6* having an especially important status. Two core genes in the brown module (*erg26* and *erg11*) and the *TAT* gene in the blue module were also located in the center of their respective networks.

Acetyl-CoA C-acetyltransferase (*ACAT1-b*) is the first enzyme in the Mevalonate pathway, catalyzing the conversion of acetyl-CoA to acetoacetyl-CoA. Hydroxymethylglutaryl coenzyme A (*hgsA*) is the following enzyme that catalyzes the conversion of acetoacetyl CoA to hydroxymethylglutaryl CoA, and it is also regulatory factor. These two genes are at the beginning of the upstream pathway of triterpenoid biosynthesis. Their position determines their status; as a result, their expression directly affects the amount of subsequent triterpenoid biosynthesis. Diphosphonate decarboxylase (*Mvd1*) catalyzes the conversion of 5-diphosphomevalonate to isopentenyl diphosphate. Isopentenyl diphosphate is a precursor to the addition of all isoprene compounds from beginning to end. The ramification of isopentenyl diphosphate is directly related to the biosynthesis of triterpenoid. It can be seen from the metabolic pathway diagram in KEGG, the expression of *mvd1* directly affects the amount of biosynthesized triterpenoid. The results of network analysis (Fig. 6) show that *ACAT1-b*, *hgsA*, and *mvd1* had direct correlations with the *erg6* gene of catalytic sterol synthesis at the downstream terminal. It can be seen that the expressions of these three core upstream genes that affect the biosynthesis of triterpenoid and sterols were uniformly regulated by the downstream *erg6* gene, indicating that the biosynthesis and accumulation of triterpenoid could be closely related to the biosynthesis of sterols.

Squalene monooxygenase (*SQLE*) catalyzes the conversion of squalene into 2,3-Oxidosqualene, which is the first oxidation step in phytosterol and triterpenoid biosynthesis. Subsequently, 2,3-Oxidosqualene is cycled by oxide squalene cyclase into a multicyclic triterpenoid backbone. These molecules are further oxidized by CYP450s to form triterpenoids. Finally, these triterpenoids are glycosylated by UGTs into triterpenoid saponins ^23^. In the cyclization of 2,3-Oxidosqualene, inner bonds are introduced into the main chain of 2,3-Oxidosqualene to form polycyclic molecules ^37^. In the process of cyclization, more than 100 triterpene backbones can be generated due to various possible combinations of inner bonds. However, only a few cyclized products are further oxidized by cyp450 ^38^. In addition, the cycled products usually have different conformations and can produce different triterpenoid saponins ^39^. *SQLE* is one of the key enzymes that regulate the biosynthesis of downstream triterpenoids and phytosterols ^40^. In study of Han et al. ^40^, two *SQLE* enzyme genes were cloned from ginseng, among which the *SQLE1* gene was interfered with to reduce ginsenoside production, and the upregulation of *SQLE2* led to enhanced phytosterol accumulation. *SQLE1* regulates the biosynthesis of ginsenoside, but not phytosterol. 2,3-Oxidosqualene is a common precursor of phytosterol and triterpenoid saponins biosynthesis. This indicates that 2,3-Oxidosqualene from the catalysis of different *SQLE* genes may be converted into different products due to the differences in conformation. In the present study, only one *SQLE* gene was annotated, which was highly expressed in the low-yielding strain. This result suggests that it may be regulated by multiple levels of post-transcriptional translation or post-translational modification. Correlation analysis showed that *SQLE* expression was mainly regulated by direct and indirect interactions of *erg6*, as well as by *msp1* and five protease genes. Furthermore, the accumulation of triterpenoid could be closely related to the biosynthesis of sterols.

Sterol 24-methyltransferase (*erg6*) catalyzes the conversion of zymosterol into fecosterol, which is then catalyzed into ergosterol by sterol isomerase. *Erg6* is a key step in the second transmethylation of sterol synthesis. More than 10 sequences of *erg6* in different plants have been isolated and cloned, which can be divided into two families according to their amino acid sequences ^41^. At least three *erg6* sequences in *Arabidopsis thaliana* have been cloned and their functions confirmed ^42^. In the present study, three sequences were annotated to *erg6*, and they were all highly expressed in the low-yielding strain. Two of these three sequences were directly and closely related to the other four core genes in the bisque4 module, indirectly related to *ACAT1-b* through two protease genes, and indirectly related to *SQLE* through the regulatory factor *YKT6* and two protease genes. These two *erg6* genes were, respectively, affected by the regulatory factors *malA* and *CYP3A24*, as well as by multiple protease genes, showing extremely complex regulatory patterns. These results indicate that the biosynthesis of sterols plays an important role in the biosynthesis and accumulation of triterpenoid in *W. cocos*.

In fungi, the 14α-methyl group required for the biosynthesis of sterols is derived from lanosterol. Sterol 14α-demethylase (*erg11*) is a cytochrome P450 ^43^ that plays an important role in catalyzing the conversion of lanosterol to sterol, and that has been shown 14 -methyl is absent from all known functional sterols ^44^. Different *erg11* genes have different special substrates. The expression of human *CYP51* is regulated by hydroxysteroids ^45^. *Erg11* can be used as a target gene to inhibit the growth of fungi ^46^. It is a key enzyme in sterol synthesis, and the resulting sterol is an important membrane component and a precursor of hormone biosynthesis ^47^. In the present study, four genes were annotated to *erg11* and their expressions were very different. One of them belonged to the brown module and was highly expressed in the low-yielding strain. It was regulated by the regulatory factors *OPT5*, *Matk*, and *betA*, as well as by multiple protease genes.

Sterol 4α-carboxylate 3-dehydrogenase (*erg26*) catalyzes the formation of keto groups at the c-3 position and the removal of carboxylate acids from c-4. It is the key enzyme for the synthesis of sterols. The growth defects of its mutant can be made up not only by exogenous sterol supply, but also by a second mutation of the gene encoding heme biosynthetase, indicating that the accumulation of *erg26* intermediate (carboxylic acid sterol) is toxic to the growth of heme active yeast cells ^46^. *Erg26* and *erg11* can be used as target genes to inhibit fungal growth. In the present study, the expression of *erg26* was regulated by the regulators *OPT5* and *GIP*, as well as by multiple protease genes. *Erg26* and *erg11* interact indirectly through four protease genes, including regulatory factors *OPT5* and *bop1-a*. They are key enzymes in sterol synthesis, and the resulting sterol is an important membrane component and a precursor of hormone biosynthesis ^47^.

Tyrosine aminotransferase (*TAT*) is an enzyme that catalyzes the conversion of the aromatic amino acid tyrosine into 4-hydroxyphenylpyruvate. It is affected by four regulatory factors and six protease genes. In the STEM analysis of Zeng et al. ^26^, three genes (*TAT*, *erg26*, and *erg11*) were indirectly correlated through the regulatory factor *Pm20d2*. *TAT*, *erg26*, and *erg11* were all identified as core genes in two different kinds of analysis, indicating that these three genes play an important role in the biosynthesis of triterpenoids and sterols in *W. cocos*. In addition, in STEM analysis, *TAT*, *erg26*, and *ERG2* were also indirectly correlated with *norA* through the action of the protease gene; *norA* was also indirectly correlated with *TAT* in the blue module. *Pm20d2* and *norA* are regulatory factors and protease genes outside the triterpenoid synthesis pathway, and they are all closely related to core genes in the two different analysis methods.

In summary, the results of the present study show eight core genes related to the synthesis and accumulation of triterpenoid, namely, *ACAT1-b*, *hgsA*, *mvd1*, *SQLE*, *erg6*, *TAT*, *erg26*, and *erg11*, as well as multiple regulatory factors and protease genes, such as *Pm20d2* and *norA*, outside the pathway. Among the eight core genes, *erg6* in the bisque4 module is at the center of the core genes, and its expression directly affects the expression of four other core genes (*ACAT1-b*, *hgsA*, *mvd1*, and *SQLE*). In the triterpenoid synthesis-related pathway, *SQLE* in the bisque4 module, *TAT* in the blue module, *erg26* and *erg11* in the brown module, as well as *Pm20d2* and *norA* outside the pathway, are six genes that all have high correlation and connectivity in the two analysis methods. This result shows that they play an important role in the biosynthesis and accumulation of triterpenoid in *W. cocos*, and they are genes that need to be focused on in follow-up studies. It has been reported ^48^ that during the development of peas after germination, the production of β-amyrin is very active, and the biosynthesis of sterols increases after several days of germination. Although the significance of this dramatic conversion between sterol and triterpenoid synthesis is unclear, similar changes occur during the development of monocotyledons in sorghum seeds, suggesting that this may be a common phenomenon among different plant species. The results of the present study also showed that the triterpenoid in *W. cocos* are closely related to the biosynthesis of sterols.

## Conclusion

Two new findings were obtained in this study: (1) *W. cocos* triterpenoid biosynthesis is closely related to eight core genes in the triterpenoid-related metabolic pathways (*ACAT1-b*, *hgsA*, *mvd1*, *SQLE*, *erg6*, *TAT*, *erg26*, and *erg11*) as well as multiple regulatory factors, such as *Pm20d2* and *norA*, outside the pathway and protease gene expressions. (2) *W. cocos* triterpenoid biosynthesis is indeed closely related to the expression of sterol metabolic pathway genes.

## Supplementary Information

Supplementary Legends.

Supplementary Figure S1.

Supplementary Figure S2.

Supplementary Figure S3.

Supplementary Figure S4.

Supplementary Figure S5.

Supplementary Figure S6.

Supplementary Figure S7.

Supplementary Figure S8.

Supplementary Figure S9.

Supplementary Figure S10.

Supplementary Table S1.

## Data Availability

The datasets generated for this study can be found in the NCBI BioProject PRJNA552734.
