# Supplementary material for: Analysis of weighted gene co-expression network of triterpenoid-related transcriptome characteristics from different strains of Wolfiporia cocos
Source: Sci Rep. 2021 Sep 14;11:18207. doi: 10.1038/s41598-021-97616-6 (PMC8440546; doi:10.1038/s41598-021-97616-6)
Supplement: Supplementary file 1 — Supplementary Legends. [file 41598_2021_97616_MOESM1_ESM.pdf]

Figure S1. Total triterpenoid content at different culture times in high-yielding (H) and low-yielding (L) strains. Red letters indicate significant differences in least-significant difference (LSD) detection at the same time point between the two strains. Blue letters indicate significant differences between time points for the same strain. Capital letters indicate highly significant differences, and lowercase letters indicate significant differences.

Figure S2. Correlation between RT-qPCR and RNA sequencing of 12 genes. Each point represents a multiple of the expression level at d 34 or d 51, at d 17 or d 34. Change in power is log base 10 transformation.

Figure S3. 12 genes expression level of RT-qPCR and RNA sequencing.

Figure S4. Correlation heat diagram of two modules. Red represents positive correlation, green represents negative, value in the module is correlation coefficient, and value in brackets is the *p*-value of correlation.

Figure S5. GO enrichment diagrams of three module genes. (a) GO enrichment diagram of bisque4 module; (b) GO enrichment diagram of blue module; (c) GO enrichment diagram of brown module.

Figure S6. GO enrichment diagrams of triterpenoid biosynthesis related genes of three modules. (a) GO enrichment diagram of bisque4 module; (b) GO enrichment diagram of blue module; (c) GO enrichment diagram of brown module.

Figure S7. Network diagram of genes related to triterpenoid biosynthesis in the brown module. Square represents the gene is triterpenoid-related genes and regulatory factors, diamond represents triterpenoid-related genes, parallelogram represents regulatory factors, circle represents protease gene. Name or number of gene marked on the node graph. Red represents the high connectivity value of genes in the module, green represents the low connectivity value, thick line between two genes represents high connectivity value between two genes, and thin line represents low connectivity value. (Cytoscape3.7.1: https://cytoscape.org/)

Figure S8. Network diagram of genes related to triterpenoid biosynthesis in the blue module. Square represents the gene is triterpenoid-related genes and regulatory factors, diamond represents triterpenoid-related genes, parallelogram represents regulatory factors, circle represents protease gene. Name or number of gene marked on the node graph. Red represents the high connectivity value of genes in the module, green represents the low connectivity value, thick line between two genes represents high connectivity value between two genes, and thin line represents low connectivity value. (Cytoscape3.7.1: https://cytoscape.org/)

Figure S9. Network diagram of genes related to triterpenoid biosynthesis in the bisque4 module. Square represents the gene is triterpenoid-related genes and regulatory factors, diamond represents triterpenoid-related genes, parallelogram represents regulatory factors, circle represents protease gene. Name or number of gene marked on the node graph. Red represents the high connectivity value of genes in the module, green represents the low connectivity value, thick line between two genes represents high connectivity value between two genes, and thin line represents low connectivity value. (Cytoscape3.7.1: https://cytoscape.org/)

Figure S10. Standardized heat map of genes in Figure 6. (Graph Pad Prism7.0: https://www.graphpad.com/)
