# Supplementary figures and images for: Analysis of weighted gene co-expression network of triterpenoid-related transcriptome characteristics from different strains of Wolfiporia cocos
Source: Sci Rep. 2021 Sep 14;11:18207. doi: 10.1038/s41598-021-97616-6 (PMC8440546; doi:10.1038/s41598-021-97616-6)

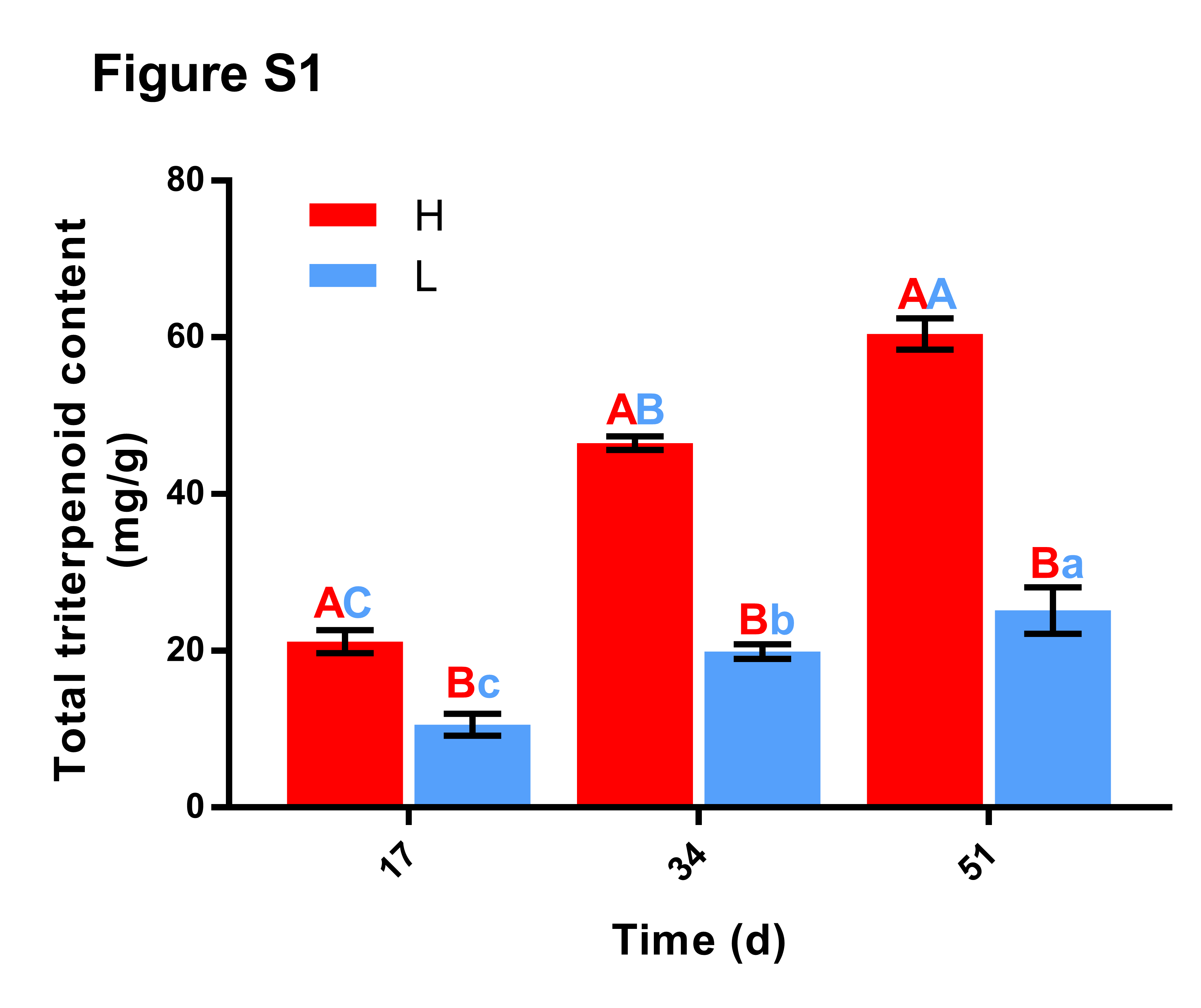

Supplement: Supplementary file 2 — Supplementary Figure S1. [file 41598_2021_97616_MOESM2_ESM.tif]

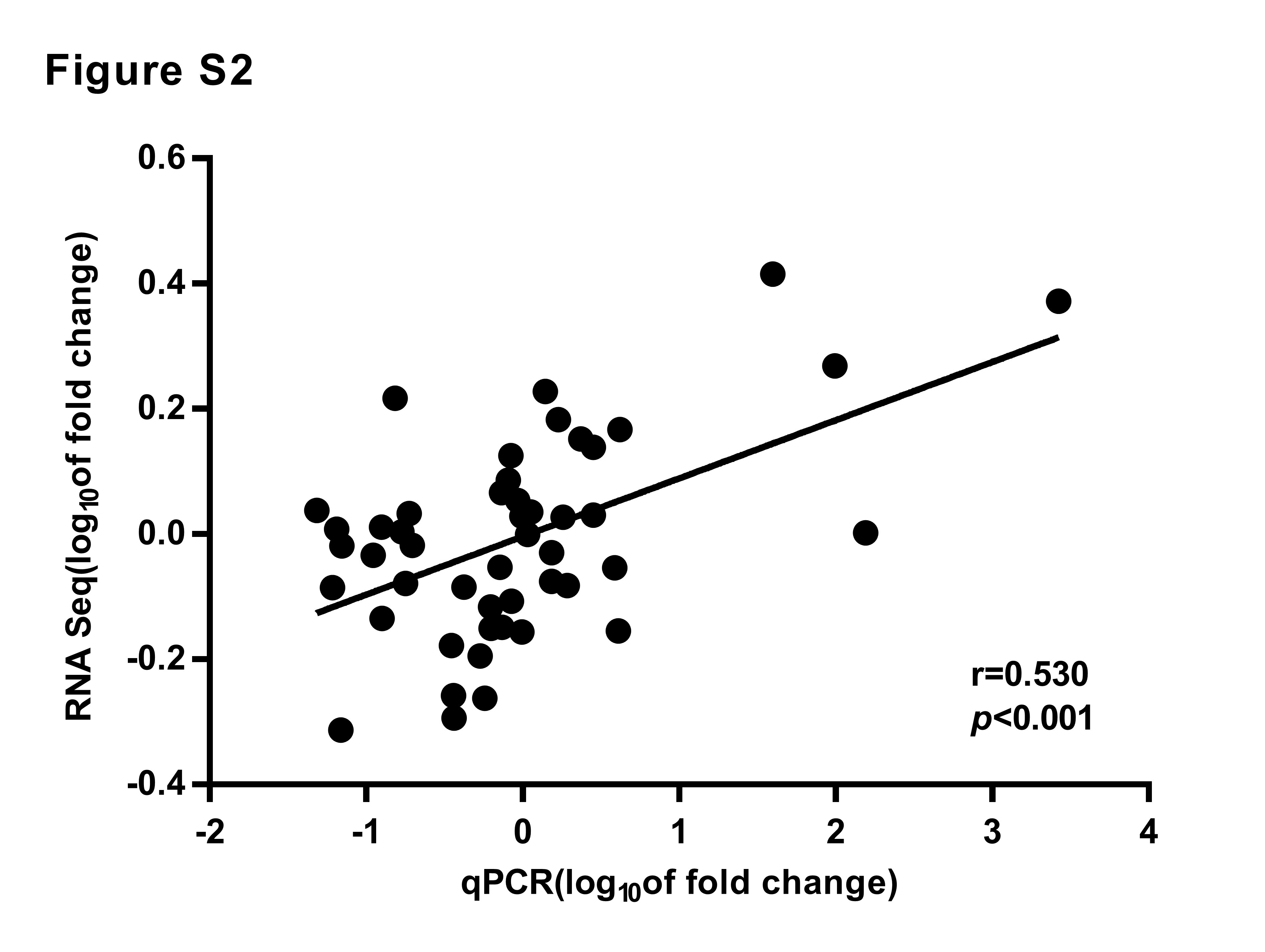

Supplement: Supplementary file 3 — Supplementary Figure S2. [file 41598_2021_97616_MOESM3_ESM.tif]

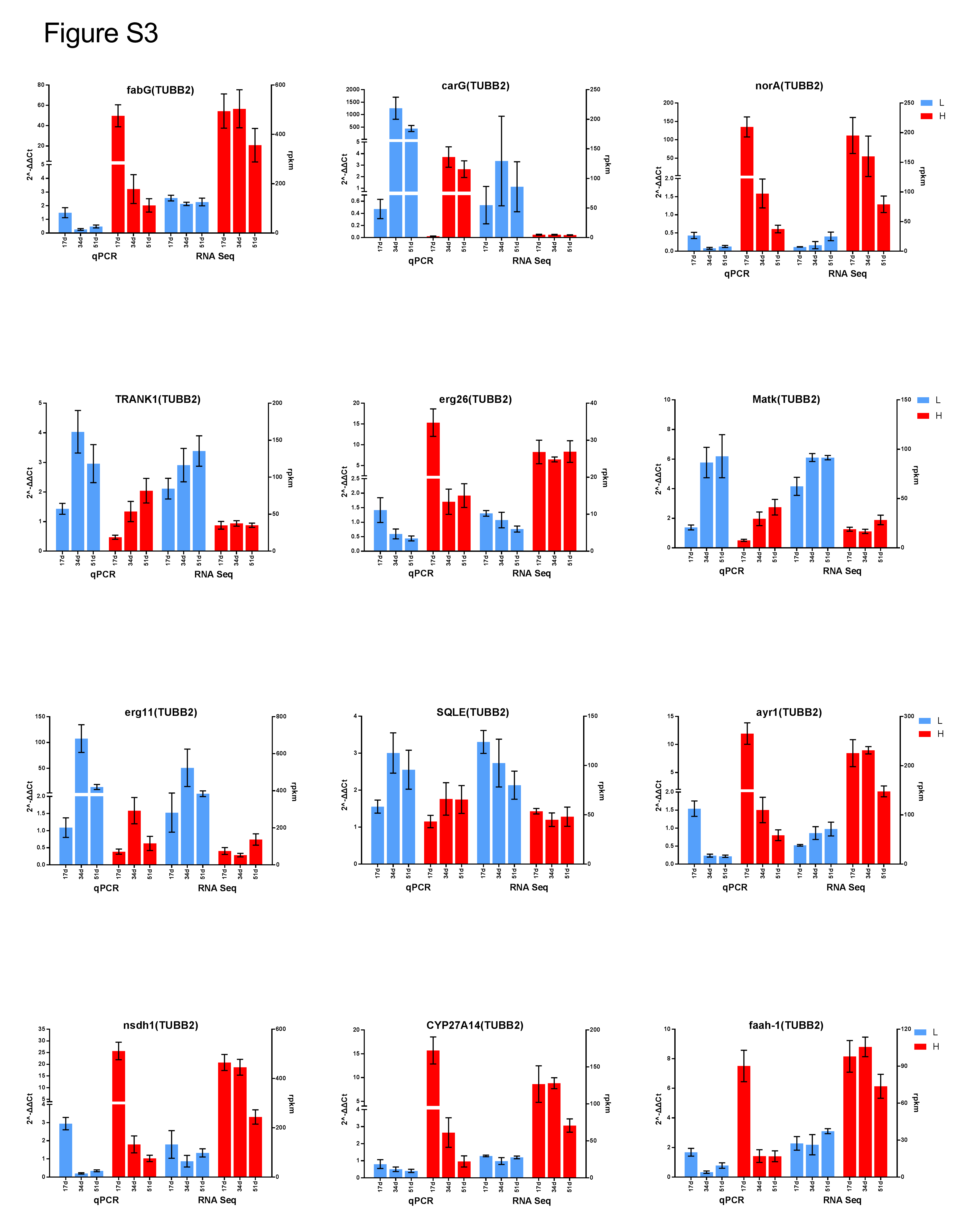

Supplement: Supplementary file 4 — Supplementary Figure S3. [file 41598_2021_97616_MOESM4_ESM.tif]

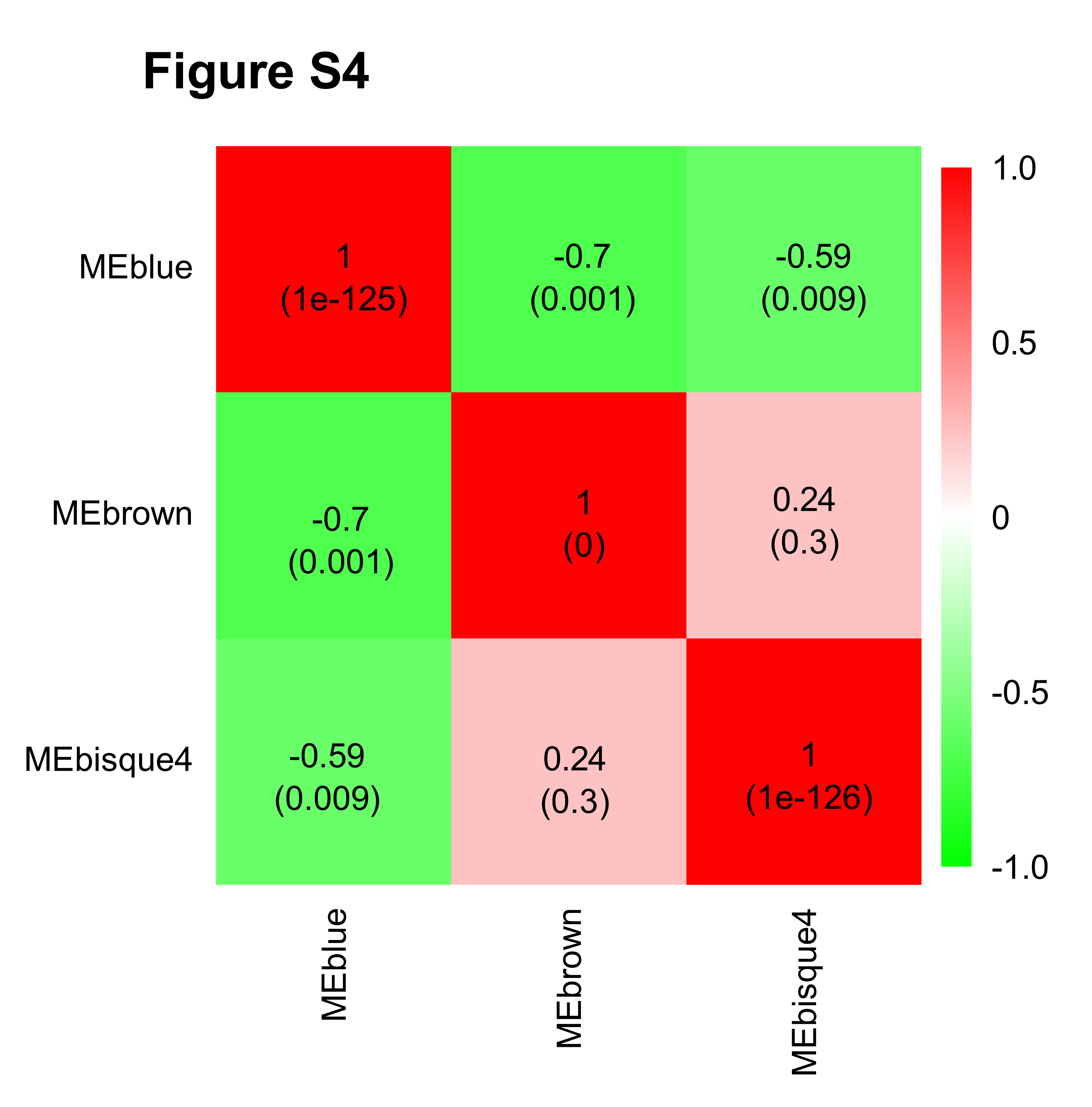

Supplement: Supplementary file 5 — Supplementary Figure S4. [file 41598_2021_97616_MOESM5_ESM.tif]

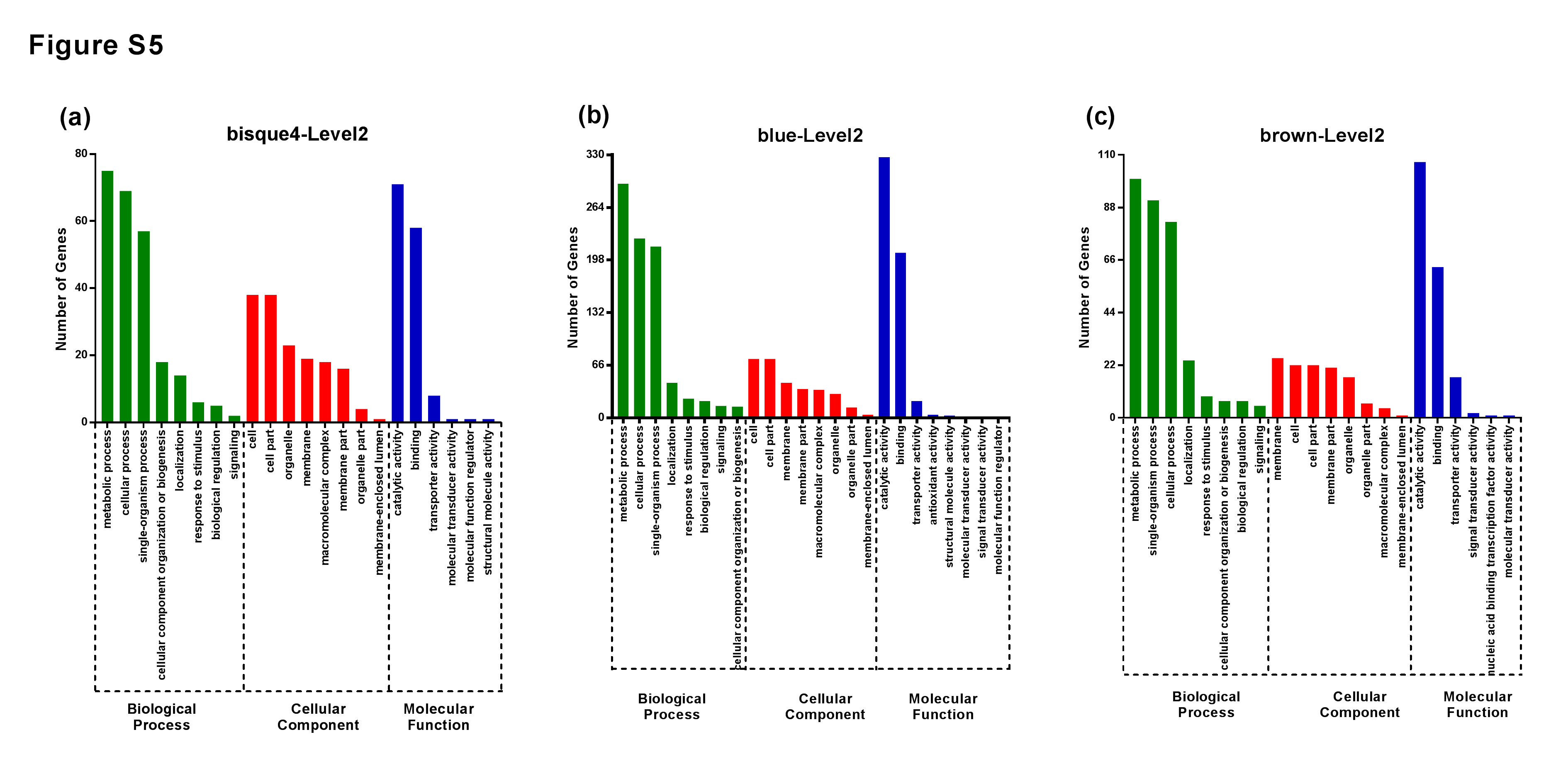

Supplement: Supplementary file 6 — Supplementary Figure S5. [file 41598_2021_97616_MOESM6_ESM.tif]

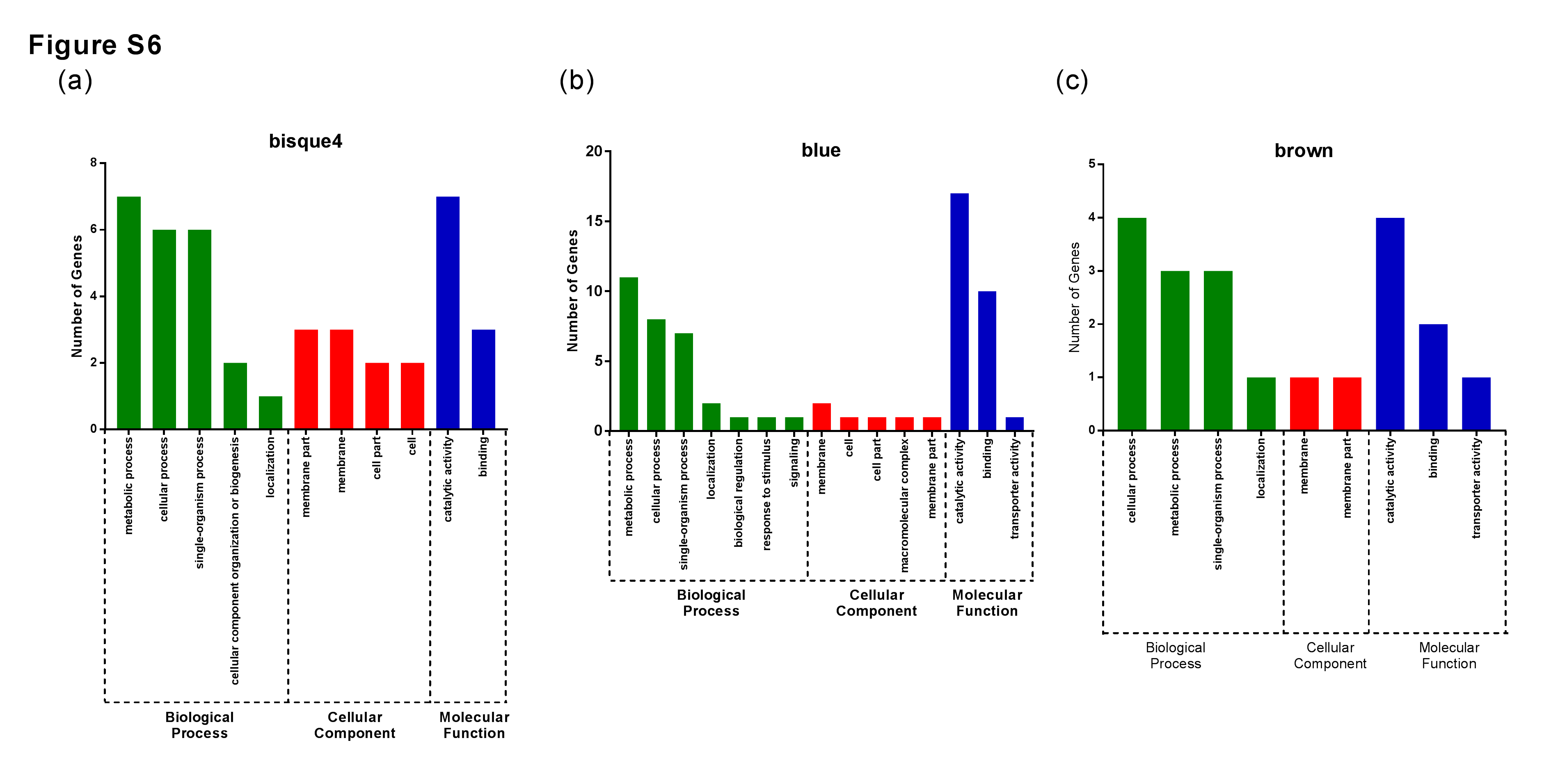

Supplement: Supplementary file 7 — Supplementary Figure S6. [file 41598_2021_97616_MOESM7_ESM.tif]

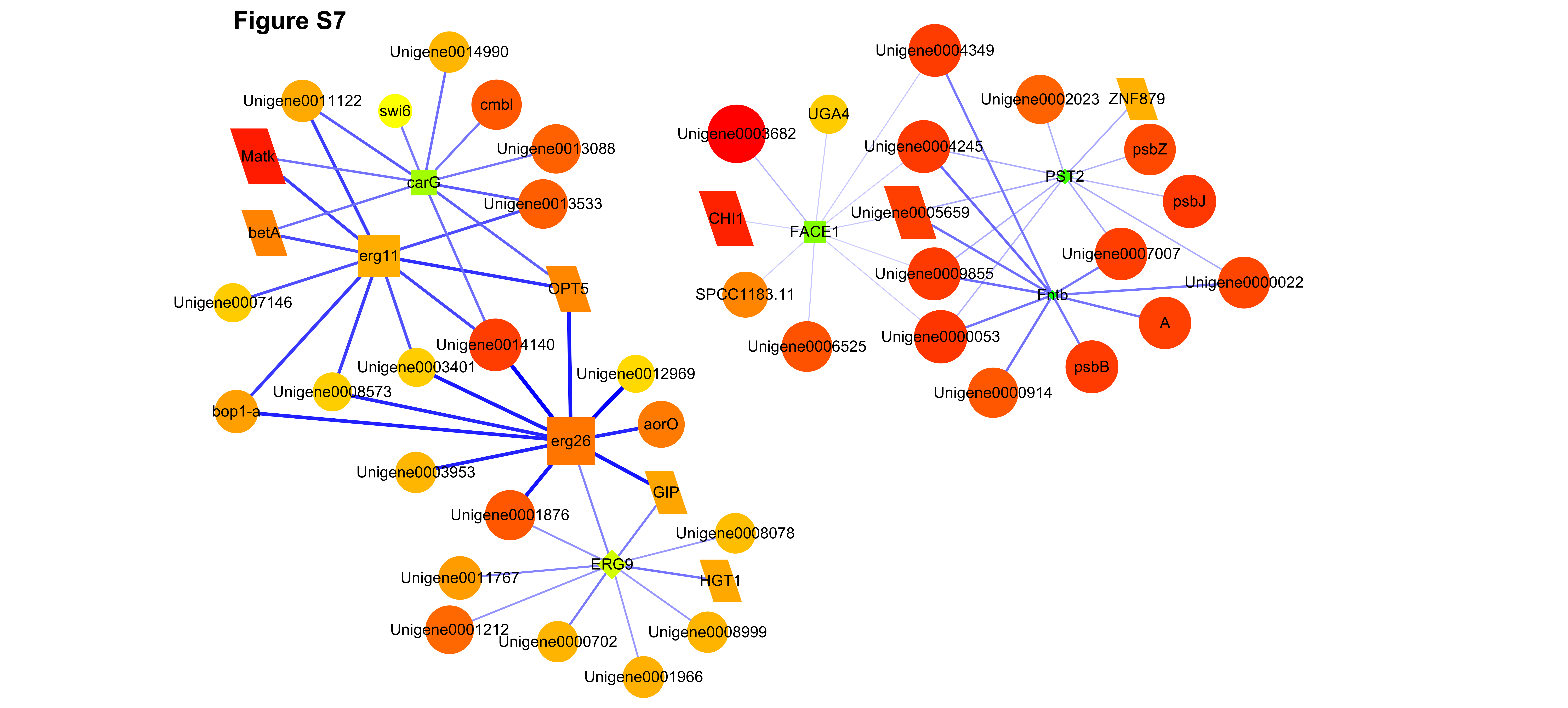

Supplement: Supplementary file 8 — Supplementary Figure S7. [file 41598_2021_97616_MOESM8_ESM.png]

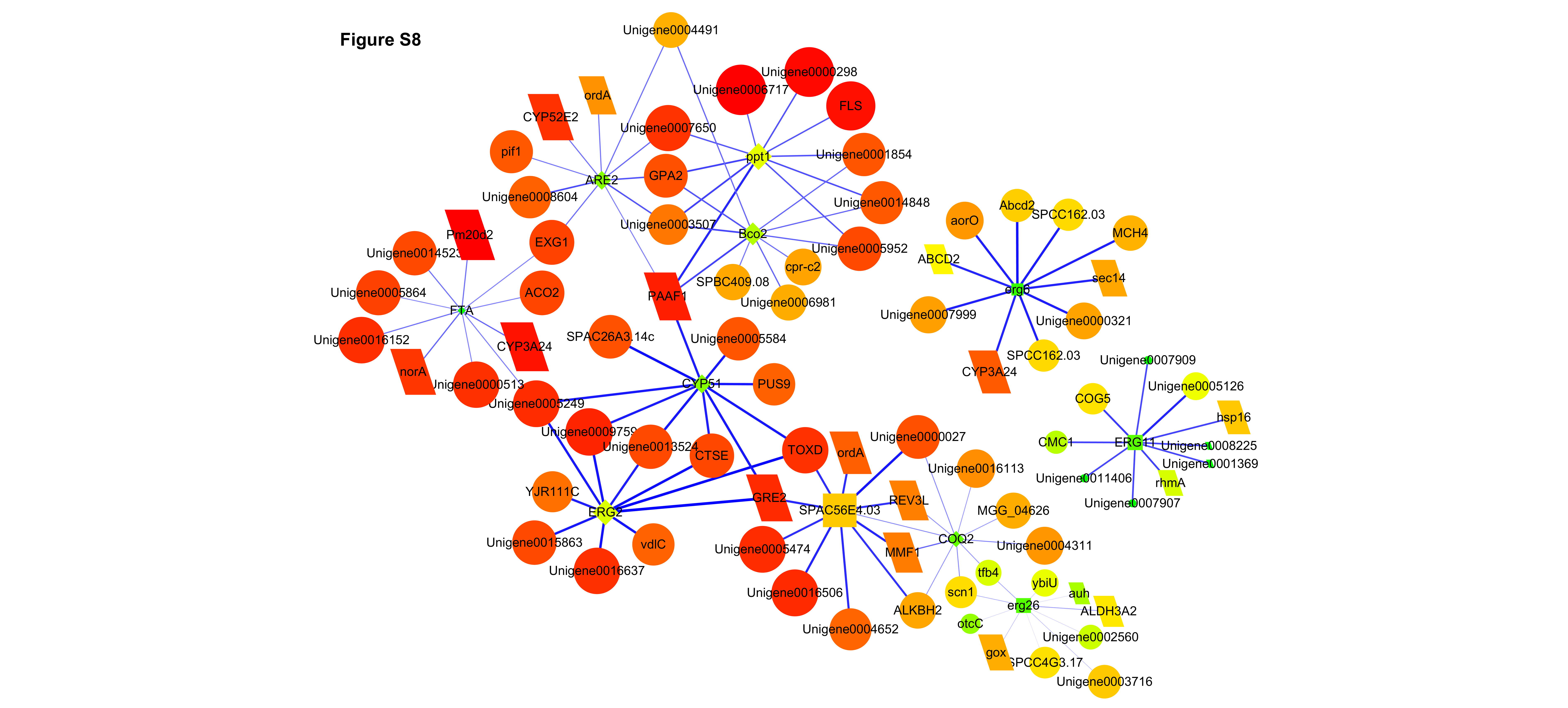

Supplement: Supplementary file 9 — Supplementary Figure S8. [file 41598_2021_97616_MOESM9_ESM.png]

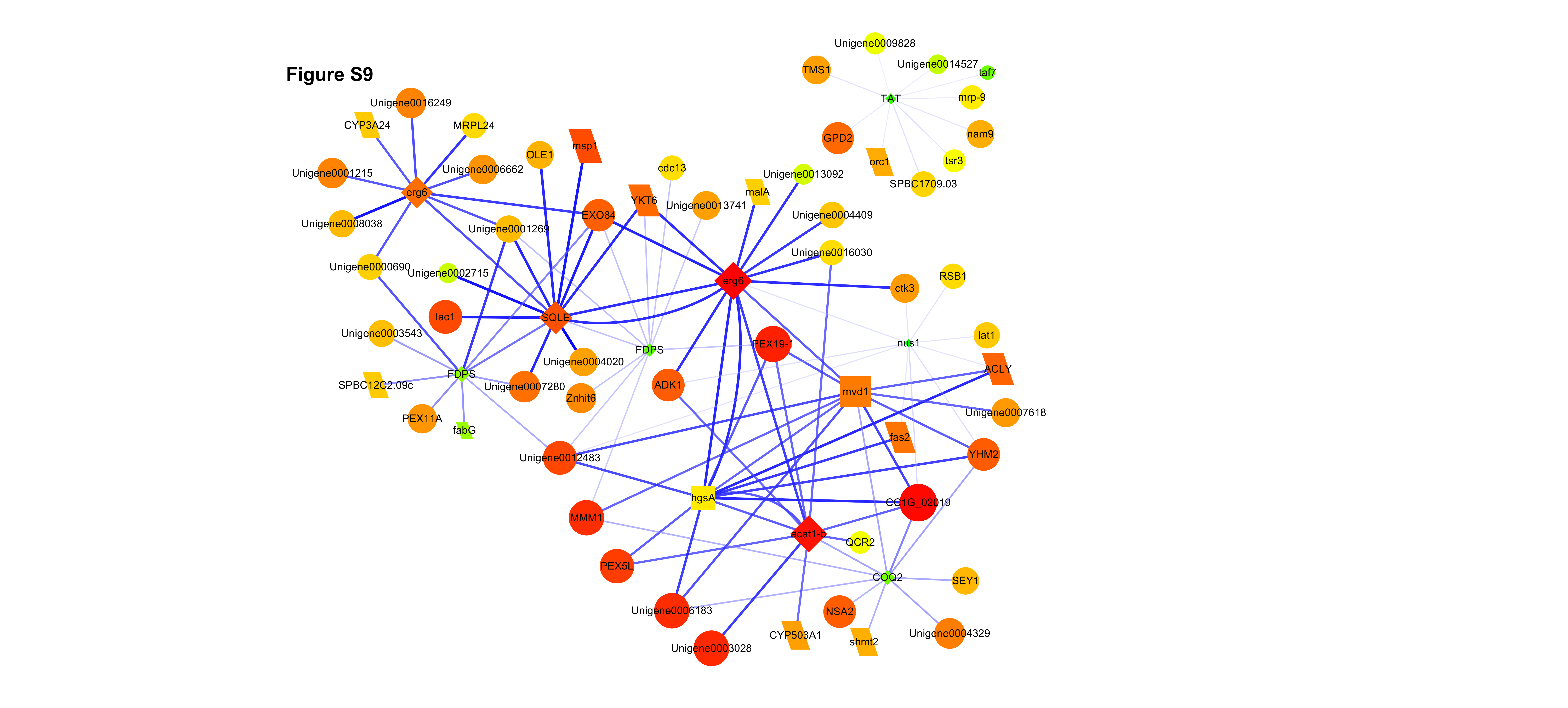

Supplement: Supplementary file 10 — Supplementary Figure S9. [file 41598_2021_97616_MOESM10_ESM.png]

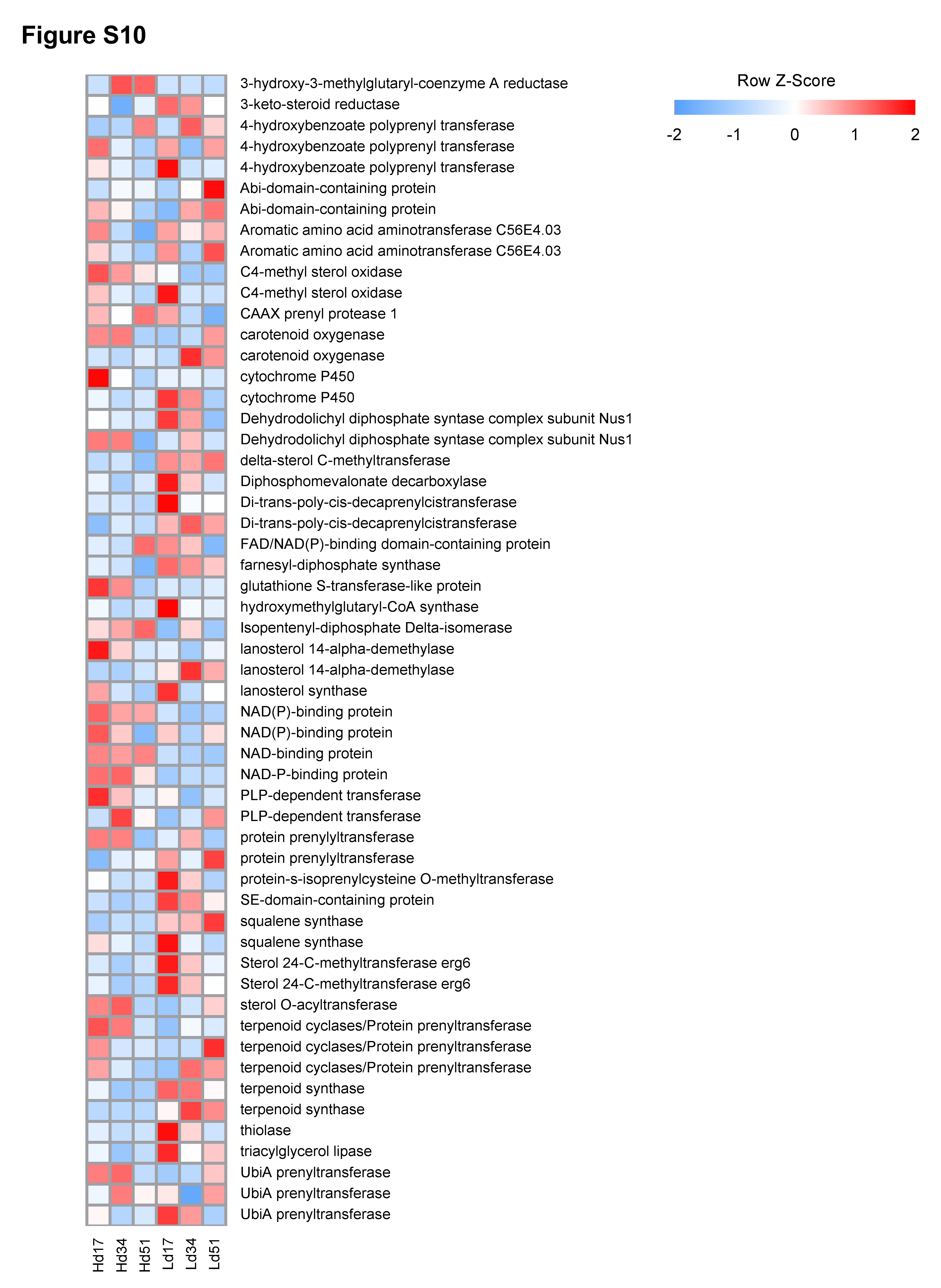

Supplement: Supplementary file 11 — Supplementary Figure S10. [file 41598_2021_97616_MOESM11_ESM.tif]
